# Exceptional and Sustained Response to Belzutifan in Von Hippel–Lindau Disease-Associated Central Nervous System Hemangioblastoma

**DOI:** 10.7759/cureus.52979

**Published:** 2024-01-26

**Authors:** Mousa Thalji, Vamshi Vadlapatla, Nicholas G Avgeropoulos, Naren Ramakrishna, Said Baidas

**Affiliations:** 1 Hematology/Oncology, Orlando Regional Medical Center, Orlando, USA; 2 Neuro-Oncology, Orlando Regional Medical Center, Orlando, USA; 3 Radiation Oncology, Orlando Regional Medical Center, Orlando, USA

**Keywords:** hif-2 alpha inhibitor, hif2α, renal cell carcinoma (rcc), pheochromocytoma, targeted therapeutics, cns hemangioblastoma, belzutifan, vhl-related syndrome, cerebellar hemangioblastoma, von hippel-lindau disease (vhl)

## Abstract

Von Hippiel-Lindau (VHL) disease is a rare genetic disorder characterized by a variety of benign and malignant neoplastic growths arising in multiple different organ systems. About 60%-84% of patients develop hemangioblastomas, benign tumors comprised of newly formed blood vessels that often occur in the central nervous system (CNS) and retinas. Treatment options for this disease were limited before the Food and Drug Administration (FDA) approval of belzutifan, a HIF2α inhibitor. We present a case of a 25-year-old woman with VHL who underwent treatment with belzutifan over 18 months. It was noted that her CNS lesions decreased significantly in size over the course of her treatment, and she had minimal adverse effects. Her excellent and sustained therapeutic response to the treatment highlights the real-world clinical benefit of belzutifan and the possibility that this could play a crucial role in treating VHL by postponing or completely avoiding repeated surgical and radiotherapeutic intervention and their associated comorbidities.

## Introduction

Von Hippel-Lindau (VHL) disease is a rare autosomal dominant condition associated with a variety of benign and malignant neoplasms such as clear cell renal carcinomas, central nervous system (CNS) hemangioblastomas, and pancreatic tumors [1]. Hemangioblastomas, which often develop in the cerebellum, spinal cord, or retina, are the most common lesions linked to VHL disorder and affect 60%-84% of patients [1,2]. CNS hemangioblastoma can present as multiple lesions rather than a solitary lesion. Given their location and multiplicity, they can lead to significant neurological compromise leading to life-threatening presentations [1].

Pathogenic germline mutations in the VHL gene on chromosome 3 are the underlying etiology of this disorder. Under normal conditions, the VHL protein functions as E3 ubiquitin ligase, which catalyzes the degradation of hypoxia-induced factors (HIF) in an oxygen-dependent mechanism. Mutations in the VHL gene lead to reduced VHL protein activity, resulting in elevated levels of HIF. These transcription factors play a major role in multiple cellular pathways, facilitating the gene expression of vascular endothelial growth factor (VEGF), platelet-derived growth factor (PDGF)β, and transforming growth factor (TGF)α. These are thought to be the factors for angiogenesis that drive the malignancy in VHL-mutated patients. With a better understanding of the molecular pathogenesis in VHL-associated neoplasms, efforts have focused on targeted systemic therapy to inhibit HIF-mediated transcriptional pathways [1].

Belzutifan has been approved by the United States Food and Drug Administration (FDA) for use in adult VHL disease patients who do not require immediate surgery and need therapy for associated renal cell carcinoma, CNS hemangioblastomas, or pancreatic neuroendocrine tumors. Belzutifan remains the only approved systemic targeted therapy in this setting [1-4].

In this article, we present a case of an exceptional clinical response to belzutifan in a patient with VHL disease and multifocal CNS hemangioblastomas.

## Case presentation

A 25-year-old woman was diagnosed with VHL syndrome at the age of 16. She presented with left arm weakness and numbness after an associated fall. Initial evaluation led to the finding of multiple intramedullary and intra-axial enhancing lesions in the cervical vertebrae on magnetic resonance imaging (MRI) as well as multiple renal, hepatic, and pancreatic lesions.

Due to the neurological symptoms at the initial presentation, she underwent her first laminectomy and resection of multiple tumors at C1, C2, and C3 levels. Pathology reports subsequently revealed hemangioblastoma, World Health Organization (WHO) grade 1. Genetic testing confirmed the diagnosis of VHL syndrome with a heterozygous deletion of exons 2-3 in the VHL gene. Two years after her initial laminectomy, she underwent another laminectomy with subtotal resection of intradural intramedullary spinal cord tumor at T10 and T11. Six months later, she underwent complete resection of the residual lesions. This led to the patient having three spinal surgeries in less than three years.

**Figure 1:**
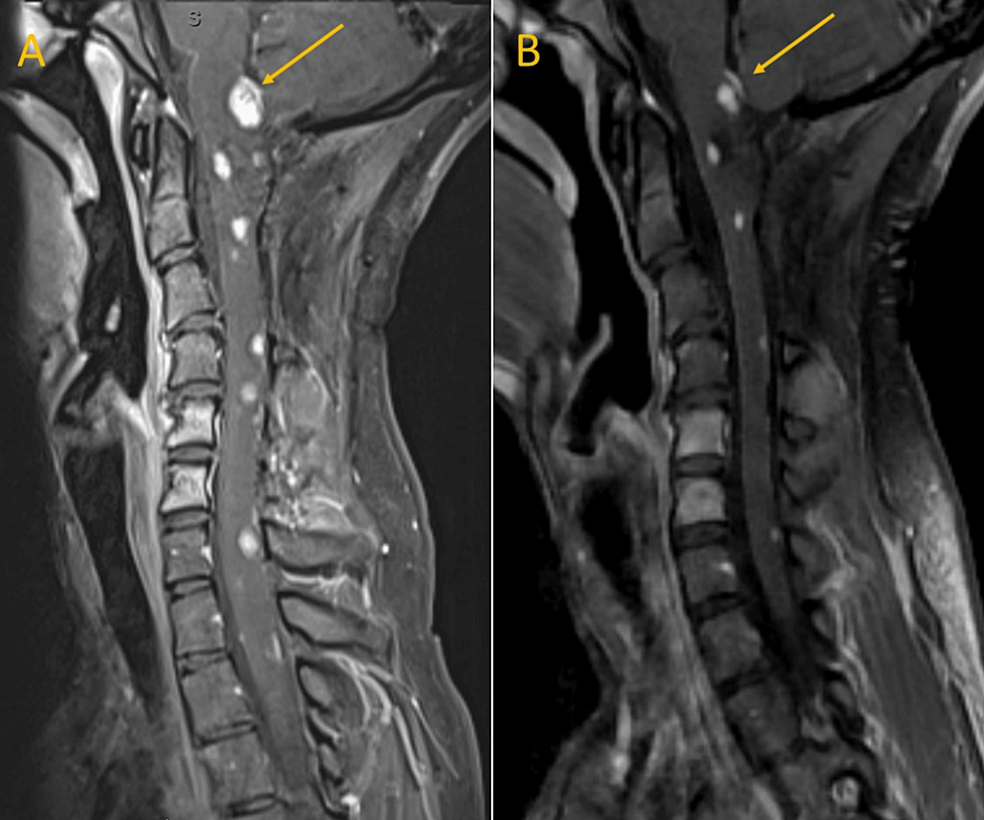
Initial magnetic resonance imaging (MRI) of the cervical spine (T1 post-contrast) (A) Surveillance MRI showing multiple enhancing lesions throughout the neuraxis with the largest lesion at the cervicomedullary junction measuring 9 x 9 x 12 mm (yellow arrow). (B) Three-month follow-up MRI post the belzutifan treatment showing an interval decrease in size to 5 x 7 x 9 mm (yellow arrow).

Over the years, she remained clinically stable and without any further neurological symptoms. Despite clinical stability, her surveillance MRIs conducted three years post surgery revealed progression in multiple enhancing lesions throughout the neuraxis, with critical enlargement of the lesion, at least four-fold in volume, at the cervicomedullary junction (9 x 9 x 12 mm) along with extensive peritumoral edema (Figure 1, Panel A). She noticed increased weakness in the right calf and a mild increase in right arm heaviness. Neurosurgery, stereotactic radiosurgery (SRS), and proton therapy were considered as potential treatment options. The patient was hesitant to undergo another surgical procedure and radiation treatment. While feasible, these options carried a significant risk of post-treatment peritumoral edema. It was then recommended to consider systemic therapy options. Therefore, after consideration of available therapies, she was started on the recently FDA-approved HIF-2 α inhibitor belzutifan.

Three months after the initiation of belzutifan, surveillance MRI of the brain and spine showed a significant interval decrease in the size of lesions throughout her cervical, thoracic spine, and cerebellum. The cervicomedullary junction mass decreased from 9 x 9 x 12 mm to 5 x 7 x 9 mm in three months with an associated decrease in the peritumoral edema (Figure 1, Panel B).

**Figure 2:**
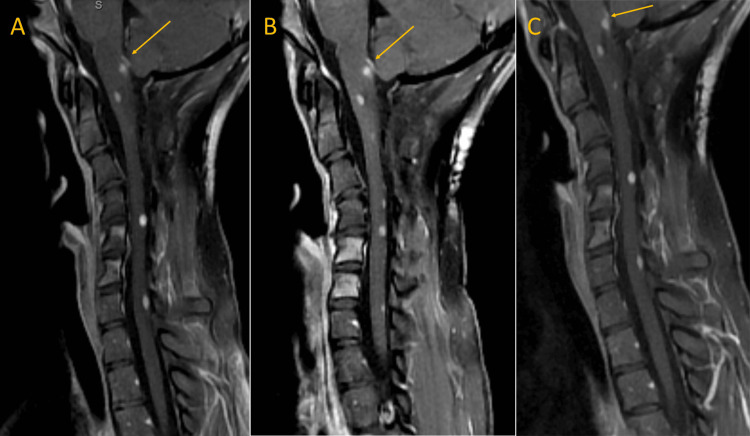
Follow-up magnetic resonance imaging (MRI) of the cervical spine at 6, 9, and 12 months, demonstrating a sustained radiologic response (T1 post-contrast) (A) Six-month cervical MRI. (B) Nine-month cervical MRI. (C) 12-month cervical MRI.

**Figure 3:**
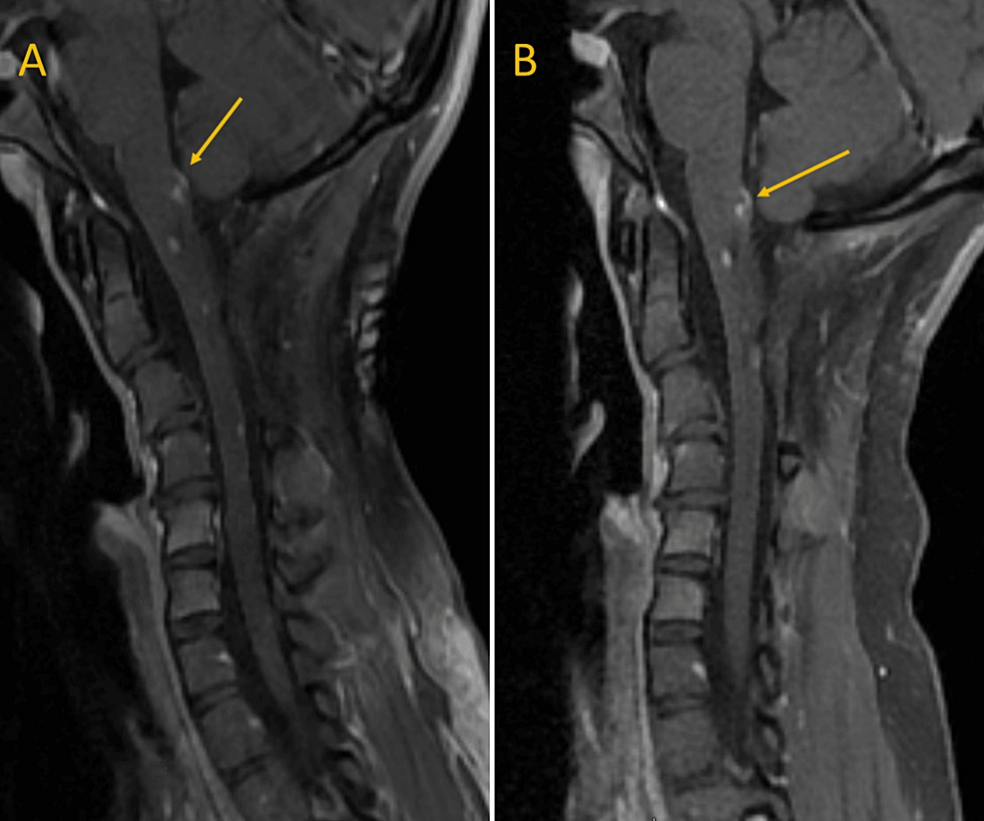
The 15- and 18-month follow-up magnetic resonance imaging (MRI) of the cervical spine (T1 post-contrast) (A) The 15-month follow-up MRI of the cervical spine. (B) An 18-month follow-up MRI of the cervical spine evidenced a sustained radiologic response.

**Figure 4:**
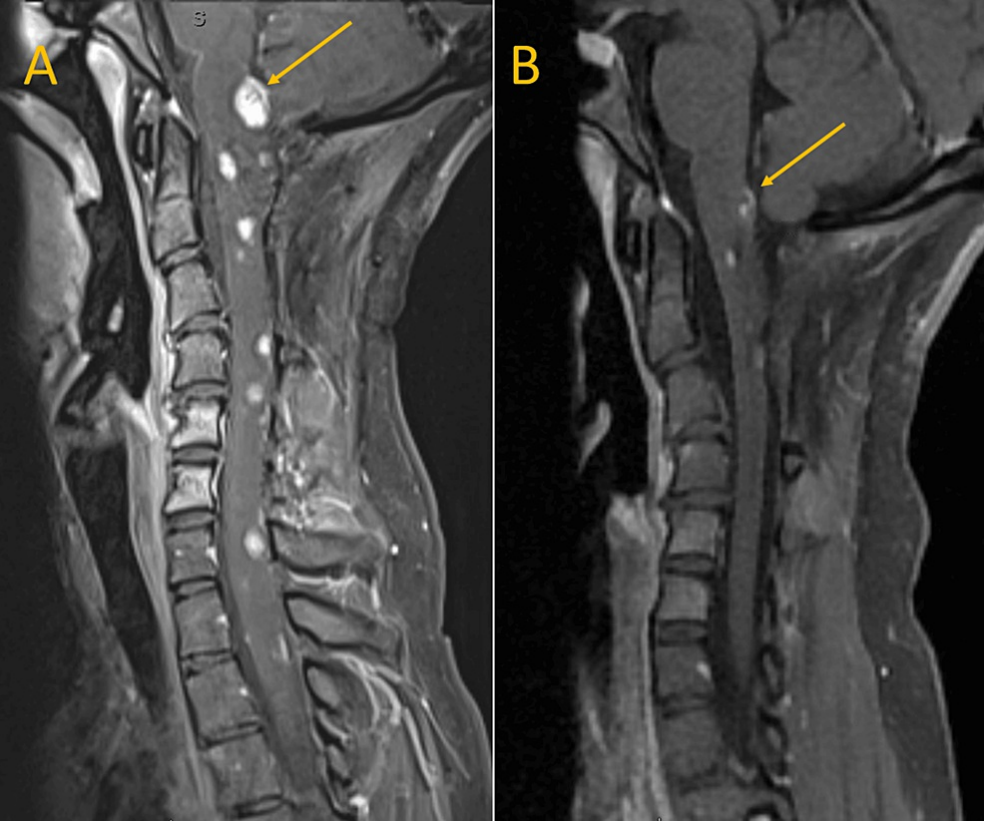
Comparison of patient's initial magnetic resonance imaging (MRI) of the cervical spine versus at 18-month follow-up (T1 post-contrast) (A) Surveillance MRI showing multiple enhancing lesions throughout the neuraxis with the largest lesion at the cervicomedullary junction measuring 9 x 9 x 12 mm (yellow arrow). (B) An 18-month follow-up MRI post the belzutifan treatment showing a durable clinical response (yellow arrow).

The patient had minor side effects of anemia with hemoglobin levels ranging between 10 and 11 g/dl and mild fatigue. She monitored her oxygen saturation via home pulse oximetry and observed that she was never hypoxic. Over the course of one year and five months, there was a decrease in the size of the posterior fossa enhancing lesions in the left cerebellum and dorsal cervical medullary junction. The cervicomedullary lesion measured 2 x 4 x 8 mm on 15-month surveillance imaging and 2 x 5 x 8 mm on 18-month imaging indicating a durable radiological response (Figures 2, 3, 4). The patient continues to tolerate this therapy well, experiencing only mild anemia and fatigue with an excellent and sustained radiological response.

## Discussion

Surgical resection, stereotactic radiosurgery, and fractionated stereotactic radiotherapy can provide excellent local control rates in the treatment of VHL-associated hemangioblastomas. However, these treatments are not without risks, including the inherent risk of surgical complications and the well-known radiation therapy risks such as radiation toxicity, peritumoral edema, radiation necrosis, etc. [1,3-6].

In a landmark clinical study, a second generation HIF2α inhibitor, belzutifan, was investigated in an open-label clinical trial in 61 patients with VHL-associated renal cell carcinoma diagnosed based on a VHL germline alteration with at least one measurable solid tumor localized to the kidney [4]. Enrolled patients had other VHL-associated tumors, including CNS hemangioblastomas in 24 patients and pancreatic neuroendocrine tumors in 47 patients. An overall response rate of 49% was noted in patients with VHL-associated renal cell carcinoma with 56% of responders having a response duration of more than 12 months. In the subset of patients with measurable CNS hemangioblastomas, there was an overall radiographic response rate of 63% (15/24), with three patients having complete response. About 73% of the responders had a response duration of more than 12 months. There is no consensus on the optimal use and sequencing of belzutifan therapy in patients with VHL-associated hemangioblastomas [1]. Belzutifan may play a crucial role by postponing or avoiding repeated surgical interventions and the associated comorbidities.

The safety profile of belzutifan is encouraging as associated adverse events are mainly low-grade. Anemia is the most common side effect reported in 90% of the patients [4]. Anemia can be attributed to the inhibition of HIF-2α, which plays a crucial role in erythropoiesis [4]. A total of five patients (8%) enrolled in the clinical study required blood transfusions. Twelve patients (20%) received erythropoiesis-stimulating agents. The second most common side effect was fatigue (66%) with hypoxia also being reported as an adverse event. However, only one patient out of the trial had grade 3 transient hypoxia without requiring supplemental oxygen. Only one out of 61 patients discontinued the therapy due to an adverse event (grade I dizziness). One patient discontinued therapy due to disease progression [1-6].

Our case demonstrates an exceptional response to belzutifan with significant radiographic response after three months of initiating therapy, which is consistent with the median time to response reported in the clinical trial [1]. She continues to have a sustained durable response of more than 18 months, which is consistent with the duration of response reported in the clinical trial. The patient had minimal side effects of mild anemia and fatigue, which did not require any interventions. Our case demonstrates the role of belzutifan in avoiding surgical or radiological interventions. Belzutifan led to a substantial and sustained clinical and radiological response. Our patient continues to tolerate this therapy well and maintains functional independence.

## Conclusions

VHL disease is a well-known albeit rare clinical entity with limited treatment options. Until recently, the standard of care was close observation with radiation therapy and/or surgical resection when clinically feasible and indicated. With the advent of belzutifan, we suspect that surgical and radiotherapy interventions can be delayed or omitted in a subset of patients who are candidates for belzutifan. Our case report demonstrates the real-world benefits of this targeted therapeutic with an excellent and sustained clinical and radiological response with a manageable side effect profile.
